# Case Report: Acquired Haemophilia A Following mRNA-1273 Booster Vaccination Against SARS-CoV-2 With Concurrent Diagnosis of Pleomorphic Dermal Sarcoma

**DOI:** 10.3389/fimmu.2022.868133

**Published:** 2022-04-11

**Authors:** Marlene Plüß, Christina Mitteldorf, Christoph Johannes Szuszies, Björn Tampe

**Affiliations:** ^1^ Department of Nephrology and Rheumatology, University Medical Center Göttingen, Göttingen, Germany; ^2^ Department of Dermatology, Venereology and Allergology, University Medical Center Göttingen, Göttingen, Germany; ^3^ Department of Hematology and Medical Oncology, University Medical Center Göttingen, Göttingen, Germany

**Keywords:** SARS-CoV-2 vaccination, booster vaccination, mRNA-1273, acquired haemophilia A, factor VIII autoantibodies, pleomorphic dermal sarcoma

## Abstract

While the global pandemic caused by severe acute respiratory syndrome coronavirus type 2 (SARS-CoV-2) is still ongoing and new virus variants are emerging, there is a universal need for vaccines to protect individuals from severe complications and ideally control the pandemic by enabling herd immunity. Several vaccines against SARS-CoV-2 have been approved and are widely used to stem the recurring waves of coronavirus disease 2019 (COVID-19). Post-marketing surveillance is essential to record even rare safety issues related to these new vaccines. Among these issues, several autoimmune phenomena have been recorded in temporal association with and feasibly triggered by a vaccination. Acquired haemophilia A (AHA) is a rare condition characterized by new-onset haemorrhagic diathesis caused by an inhibitor of blood clotting factor VIII (FVIII), often in the elderly and most commonly associated with autoimmune or malignant disease. There have been a small number of AHA cases triggered by vaccinations, including those against SARS-CoV-2. We report the first case of AHA in temporal association with an mRNA-1273 booster vaccination. The diagnosis was made promptly, and the patient received appropriate care including immunosuppression using glucocorticoids, cyclophosphamide (CYC) and rituximab (RTX). The haemorrhage ceased after escalation of treatment, and the patient is recovering. Concurrent malignancy was initially ruled out using a wide scope of diagnostic tests, but pleomorphic dermal sarcoma (PDS) of the forehead occurred after initiation of specific AHA immunosuppressive treatment. Since large vaccination programs are ongoing worldwide and potential adverse events during post-marketing surveillance have been reported following vaccination against SARS-CoV-2, this case illustrates challenges in rare events occurring in association with SARS-CoV-2 vaccination and to proof a causal relationship. Therefore, there is an urgent need for reporting any events in association with SARS-CoV-2 vaccination, but also a crucial discussion about possible concurrent triggers and follow-up information about individual patients.

## Introduction

As the coronavirus pandemic is ongoing and new variants of SARS-CoV-2 emerge, there is an urgent need for vaccines to protect individuals at high risk for complications and to potentially control disease outbreaks by herd immunity (1, 2). The European Medicines Agency (EMA) has approved the use of vaccines containing a nucleoside-modified messenger RNA (mRNA) that encodes the viral spike glycoprotein (S) of SARS-CoV-2, as well as an adenovirus-based DNA vector vaccine encoding the SARS-CoV-2 S glycoprotein. Surveillance of rare safety issues related to these vaccines is progressing, since more data emerge about adverse side effects of SARS-CoV-2 vaccines during post-marketing surveillance (3). Acquired haemophilia A (AHA) is a bleeding condition in which patients develop autoantibodies directed against clotting factor VIII (FVIII) (4). AHA is a rare disorder manifesting at a median age of 78 years and an incidence of 1.48 per million per year (5). Distinct comorbidities such as autoimmune diseases and cancers are commonly associated with AHA (6). Typically, patients with AHA present with acute or recent bleeding symptoms, without a previous history of bleeding, with laboratory investigations showing an isolated prolonged activated partial thromboplastin time (aPTT), reduced FVIII activity (<1% in 50% of cases, <5% in 75% of cases, <40% in 100% of cases), and the presence of autoantibodies detected by the Bethesda assay or by enzyme-linked immunosorbent assay (ELISA) (7, 8). Bleeding complications in AHA most commonly involve the skin, while deep tissue bleeding including haemarthrosis is more commonly observed in congenital haemophilia A (6). A few cases have been reported in which vaccines were shown to trigger AHA (9, 10). During the ongoing coronavirus pandemic, AHA has also been described as an autoimmune phenomenon associated with SARS-CoV-2 infection (11–13). Most recently, immunization with both mRNA vaccines against SARS-CoV-2 (BNT162b2 by Pfizer/BioNTech as well as mRNA-1273 by Moderna) has been reported as a trigger for AHA in single cases (14–20). However, it remains unclear if SARS-CoV-2 booster vaccination can also trigger AHA. This is especially relevant since clinicians should be aware of this rare but severe complication that requires specific diagnostic work-up and treatment. We herein provide the first report of AHA following mRNA-1273 booster vaccination against SARS-CoV-2, further complicated by a concurrent diagnosis of dermal sarcoma after initiation of immunosuppression for AHA treatment.

## Case Description

**Figure 1:**
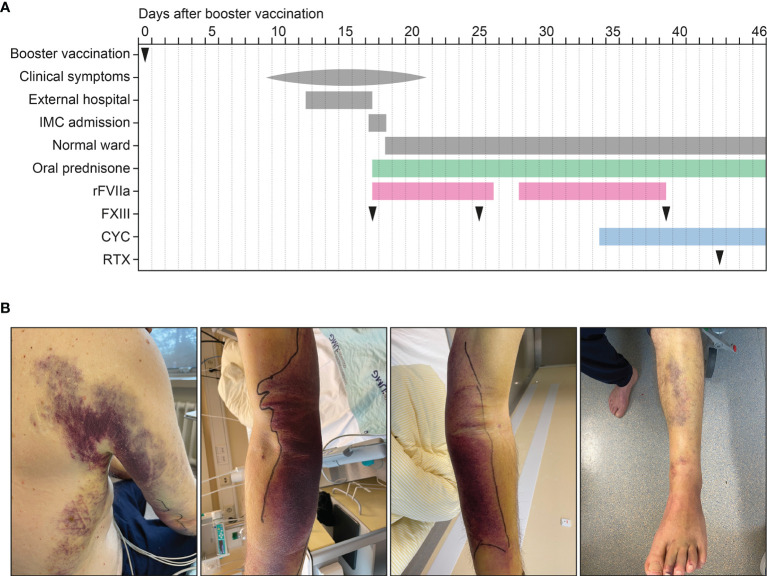
Time course of the case and clinical findings after SARS-CoV-2 booster vaccination. **(A)** Time course of booster vaccination, onset of symptoms, admission, oral prednisone, substitution of rFVIIa and FXIII, oral CYC and intravenous RTX treatment. **(B)** Ecchymosis on the upper and lower extremities and the trunk. CYC, cyclophosphamide; FXIII, factor XIII; IMC, intermediate care unit; rFVIIa, recombinant activated FVII; RTX, rituximab.

A 72-year-old Caucasian male with a past medical history of benign prostatic hyperplasia (BPH) and carpal tunnel syndrome, a 50 pack-year smoking history, and no documented history of COVID-19 received homologous BNT162b2 vaccinations (05/2021 and 06/2021) and an mRNA-1273 booster vaccination against SARS-CoV-2 (12/2021). The patient had no allergies, no history of immune deficiency, no recent infections or fevers, and no personal or family history of any bleeding disorders. Nine days after mRNA-1273 booster vaccination, the patient started to notice several bruises appearing on his arms, left leg and trunk (**Figure 1A**). All bruising was spontaneous without any trauma and were not directly observed after vaccine injection. The symptoms were accompanied by generalized weakness, myalgia, arthralgia of the shoulders, and exercise dyspnoea over the course of several days. Initially admitted to an external hospital, the patient was afebrile and haemodynamically stable. The physical exam revealed generalized pallor of the skin and mucous membranes, as well as large ecchymosis on both forearms and elbows, the right trunk and flank as well as the left leg (**Figure 1B**). The in-depth physical exam was otherwise unremarkable, with no palpable lymphadenopathy or organomegaly. The patient’s complete blood count showed anaemia (haemoglobin 7.8 g/dL, normal range: 13-18 g/dL) with normal platelets (320×10^3^/µL, normal range: 150-500×10^3^/µL) and leukocyte counts (8.25×10^3^/µL, normal range: 4-11×10^3^/µL). The coagulation profile revealed a normal international normalized ratio (INR) of 0.9 (normal range: 0.8-1.2), but a distinctly prolonged aPTT of 164.6 seconds (normal range: 25-37 seconds). Both fibrinogen (245.2 mg/dL, normal range: 200-393 mg/dL) and antithrombin III (AT III: 87%, normal range: 83-128%) were within normal limits. The patient was transfused two units of packed red cells (PRC) and then admitted to the intermediate care ward (IMC) at our tertiary centre for further treatment of what was suspected to be AHA. Upon admission, the complete blood count confirmed anaemia (Hb 8,5 g/dL, normal range: 13.5-17.5 g/dL) with normal platelets and leukocyte counts. A differential blood count showed normal distribution of lymphocytes (40%, normal range: 20-45%), monocytes (9.7%, normal range: 3-13%), eosinophils (0.5%, normal range: ≤8%), basophils (0.2%, normal range: ≤2%) and neutrophils (49.5%, normal range: 40-76%, **Table 1**), with no pathological B cell population detected by flow cytometry. Kidney and liver function tests were normal, except for a slightly elevated bilirubin level (1.4 mg/dL, normal range: 0.3-1.2 mg/dL). Lactate dehydrogenase (LDH) was mildly increased (299 U/L, normal range: 125-250 U/L), but there was no sign of intravasal haemolysis (haptoglobin 1.64 g/L, normal range: 0.14-2.58 g/L). C-reactive protein (CRP) was moderately increased at 18.5 mg/L (normal range: ≤5 mg/L). As indicated above, aPTT was severely prolonged at 130 seconds (normal range: 25-37 seconds), INR (1.0) and fibrinogen (355 mg/dL) were again within the normal range, as was prothrombin time (14.2 seconds, normal range: 10.3-16.6 seconds, **Table 1**). Differential coagulation diagnostics showed normal activity for factor II (FII: 90%, normal range: 79-131%), FV (144%, normal range: 62-139%), FVII (88%, normal range: 50-129%) and FX (87%, normal range: 77-131%) and mildly reduced activity of FIX (48%, normal range: 65-150%), FXI (35%, normal range: 65-150%) as well as FXIII (40%, normal range: 63-157%, **Table 1**). There was no measurable activity of FVIII (<0.1%, normal range: 70-170%, **Table 1**). To complete the work-up of aPTT prolongation, lupus anticoagulans and anti-cardiolipin antibodies were measured and yielded normal results (**Table 1**). As previously observed in AHA, MixCon-LA ratio was elevated because FVIII inhibitors are reported to interfere with LA assays, producing false-positive results for those tests (21, 22). Final tests revealed an elevated von Willebrand factor (vWF) activity (210%, normal range: 66-176%) and found a very high FVIII inhibitor measured at 158.6 bethesda units (BU, **Table 1**). Together with the fully eliminated FVIII activity described above, the diagnosis of AHA was confirmed.

**Table 1:** Laboratory findings after admission.

	Value	Normal range
*White blood differential*		
Leukocytes – 1,000/µL	6.62	4.0-11.0
Lymphocytes – %	40	20-45
Monocytes – %	9.7	3-13
Eosinophils – %	0.5	≤8
Basophils – %	0.2	≤2
Neutrophils – %	49.5	40-76
*Coagulation diagnostics*		
aPTT – seconds	130	25-37
INR – ratio	1.0	0.8-1.2
Fibrinogen – mg/dL	355	200-393
Thrombin time – seconds	14.2	10.3-16.6
FII – %	90	79-131
FV – %	144	62-139
FVII – %	88	50-129
FVIII – %	0.5	70-170
FIX – %	48	65-150
FX – %	87	77-131
FXI – %	35	65-150
FXIII – %	40	63-157
vWF antigen – %	210.4	66-176
Ristocetin cofactor – %	169.2	61-239
Lupus anticoagulans – ratio	1.03	0.8-1.2
MixCon-LA – ratio	1.29	0.8-1.1
Anti-cardiolopin IgG – U/mL	17	<40
Anti-cardiolopin IgM – U/mL	2.1	<40
FVIII inhibitor – BU	158.6	n.a.

aPTT, activated partial thromboplastin time; BU, bethesda units; FII, factor II; FV, factor V; FVII, factor VII; FVIII, factor VIII; FIX, factor IX; FX, factor X; FXI, factor XI; FXIII, factor XIII; INR, international normalized ratio; n.a., not available; vWF, von Willebrand Factor.

The patient was started on recombinant activated FVII (rFVIIa) and received oral prednisone (100 mg per day) as per the recommendations of the German Society for Thrombosis and Haemostasis Research (GTH) and international treatment guidelines (7, 8). A protone pump inhibitor (PPI), Vitamin D and trimethoprime sulfamethoxazole were given as adjunct treatment with the high corticosteroid doses for ulcer, osteoporosis and pneumocystis jirovecii prophylaxis, respectively. The aPTT gradually decreased with the rFVIIa treatment, which was tapered from Q3H to Q6H after four days of treatment, and after a further reduction to Q12H was discontinued after a total treatment duration of 10 days. Because of new ecchymosis within 48 hours, rFVIIa substitution was resumed at Q3H, with a careful taper after another four days and a longer course of Q12H treatment until day 23 (**Figure 1A**). FXIII was substituted three times (**Figure 1A**). The FVIII inhibitor was shown to decrease from 158.6 BU on admission to 83.2 BU on day 8 and 20.6 BU on day 21. FVIII activity increased to 2.4% on day 9, 5.9% on day 14 and up to 8% on day 29 with a spontaneous aPTT of 49 seconds at this point. Haemoglobin levels were stable and climbing, so that no blood transfusions were necessary. In view of the ongoing AHA activity and prolonged need for rFVIIa substitution, the immunosuppression with oral prednisone was continued and the patient received oral cyclophosphamide (CYC, 150 mg daily) from day 18 onwards and weekly treatment with rituximab (RTX, 375 mg/m^2^) on day 27 (**Figure 1A**).

## Diagnostic Work-Up of Concurrent Triggers for AHA

The patient underwent further diagnostic testing in order to rule out active malignancy as another potential trigger for AHA. In view of the past medical history of BPH, prostate-specific antigen was measured, which was within normal limits (3.14 µg/L, normal range: <4 µg/L) and hence gave no indication of prostatic cancer. A CT scan of the neck, chest, abdomen and pelvis was performed, which yielded a 4 cm mass on the left adrenal gland as the sole pathological finding. A dedicated adrenal CT with arterial and portalvenous contrast phase confirmed the mass to be a lipid adenoma. An MRI of the cranium was completed and showed no evidence of an intracranial lesion. During the further hospital stay, a small nodule (1 cm in diameter) was discovered on the patient’s forehead. A punch biopsy revealed a pleomorphic dermal sarcoma (PDS), and the patient was presented to the multidisciplinary tumour board at our tertiary centre. Surgical excision was recommended. The patient did present to the surgical clinic earlier than scheduled and described a now rapidly growing nodule on his forehead, so that the date of surgery had to be expedited with complete resection. In summary, we herein present the course of a patient with AHA in close temporal association with both, mRNA-1273 booster vaccination against SARS-CoV-2 as well as the diagnosis of a PDS. The case was reported to the Federal Institute for Vaccines and Biomedicines (Paul-Ehrlich-Institute). According to current recommendations, the patient will be followed-up after complete remission, monitoring FVIII activity monthly during the first 6 months, every 2-3 months up to 12 months, and every 6 months during the second year and beyond (8).

## Discussion

To our knowledge, this is the first case of AHA following mRNA-1273 booster vaccination against SARS-CoV-2. This case illustrates that a thorough diagnostic work-up for malignancy is mandatory at the diagnosis of AHA, even if other immune-mediated triggers seem equally likely. As previously observed, there is a close temporal association with symptoms starting nine days following vaccination in our case within a similar timeframe of one to three weeks (9, 10, 14–20). The patient in this case report was treated with prednisone (100 mg per day) and received CYC (150 mg per day) and RTX (375 mg/m^2^) in a slightly accelerated version of the current recommendations of the German Society of Thrombosis and Haemostasis Research (GTH). The algorithm of the GTH working group on AHA suggests corticosteroid treatment for three weeks or until partial remission (PR) is achieved, which is defined as FVIII activity >50% without blood products and with no active bleeding. If PR is not achieved, the guidelines recommend CYC (week 4-6) followed by RTX (week 7-10) (8). Our literature research revealed that most published cases of AHA after SARS-CoV-2 vaccination were treated with steroids (9 out of 10), one patient received CYC alone, one patient RTX alone, two patients combined CYC/RTX treatment and one patient azathioprine (14–20). There was one fatality after arterial bleeding from a ruptured gallbladder, all other cases of AHA in association with SARS-CoV-2 vaccination resolved or were resolving at the time of publication (14–20).

Because of the frequently observed parallel occurrence of AHA and malignancies, our patient was worked-up in-depth regarding a possible malignant trigger for AHA and specific AHA treatment with immunosuppression including steroids, CYC and RTX was initiated (6). Because initial work-up for concurrent malignancy came back negative, the working diagnosis for our patient was that of an autoimmune-mediated AHA triggered by the booster vaccination. However, five weeks after admission to our hospital, a small nodule on the forehead was biopsied to fully rule out a coexisting tumour. When histopathology confirmed PDS, the initial working diagnosis had to be partly overhauled. Interestingly, there has only been one case report of AHA with myxofibrosarcoma and one report of AHA with Kaposi’s sarcoma (23, 24). It remains unclear whether the sarcoma can be counted as a trigger for the severe AHA in the case presented here. However, sun light exposure presents a major risk factor for PDS, but immunosuppression can be an additional risk factor. The patient reported a rapid growth of the PDS after initiation of specific AHA treatment with immunosuppression including steroids, CYC and RTX (25). In addition, the question whether our patient might have developed AHA without the booster vaccination remains impossible to answer. Interestingly, a similar argument was posed after reports of three cases of AHA in close temporal association with mRNA-1273 vaccination in Switzerland (26). Using demographic data on the average incidence of AHA, and statistical methods to determine the probability of AHA and vaccination randomly coinciding, they concluded that the reported AHA incidence is “exactly as expected”, arguing against a cluster of association (26). Supporting the view that the booster vaccination can be counted as a feasible trigger, there have been a few other reports of AHA after the first or second dose of SARS-CoV-2 vaccination, as well as other autoimmune phenomena like cutaneous vasculitis, Guillan-Barré syndrome, or haematological manifestations such as immune thrombocytopenia (27–31). One possible pathophysiological mechanism of vaccine-triggered autoimmunity lies in the activation of hitherto dormant autoreactive T and B cells, as well as molecular mimicry (32). Interestingly, there have been cases of AHA after SARS-CoV-2 infection as well, indicating that the particular antigen (mainly the spike protein of SARS-CoV-2 as used in these vaccines) might play a role in this particularly powerful immune activation (11–13). This case illustrates challenges in rare events occurring in association with SARS-CoV-2 vaccination and to proof a causal relationship. Therefore, there is an urgent need for reporting any events in association with SARS-CoV-2 vaccination, but also a crucial discussion about possible concurrent triggers and follow-up information about individual patients.

## Conclusions

As wide-ranging vaccination programmes are ongoing worldwide, post-marketing surveillance is essential for the safe delivery of the different SARS-CoV-2 vaccines. The detection and transparent communication of any adverse events including rare complications is important. This is especially relevant since these unusual but severe complications require specific diagnostic work-up and treatment. This case illustrates that a thorough diagnostic work-up for malignancy is mandatory at the diagnosis of AHA, even if other immune-mediated triggers seem equally likely. Moreover, oncological follow-up should be guaranteed, seeing as the severe immunosuppressive treatment that can be required leads to an increased risk of new or newly un-masked, rapidly proliferating malignancy. Our report aims to sensitize clinicians in the field to this rare but potentially severe complication to encourage prompt recognition and diagnosis of AHA, a thorough investigation of possible concurrent triggers, as well as timely treatment once found.

## Data Availability Statement

The original contributions presented in the study are included in the article/supplementary material, further inquiries can be directed to the corresponding author.

## Ethics Statement

Ethical review and approval was not required for the study on human participants in accordance with the local legislation and institutional requirements. The patients/participants provided their written informed consent to participate in this study. Written informed consent was obtained from the individual(s) for the publication of any potentially identifiable images or data included in this article.

## Author Contributions

MP and BT conceived the case report, collected and analyzed data and co-wrote the manuscript. MP, CS, and BT were directly involved in the treatment of the patient. CM performed histological evaluation. All authors contributed to the article and approved the submitted version.

## Conflict of Interest

The authors declare that the research was conducted in the absence of any commercial or financial relationships that could be construed as a potential conflict of interest.

## Publisher’s Note

All claims expressed in this article are solely those of the authors and do not necessarily represent those of their affiliated organizations, or those of the publisher, the editors and the reviewers. Any product that may be evaluated in this article, or claim that may be made by its manufacturer, is not guaranteed or endorsed by the publisher.
